# Over-Expression of Long Non-Coding RNA-AC099850.3 Correlates With Tumor Progression and Poor Prognosis in Lung Adenocarcinoma

**DOI:** 10.3389/fonc.2022.895708

**Published:** 2022-05-11

**Authors:** Xi Chen, Jishu Guo, Fan Zhou, Wenjun Ren, Jun Pu, Luciano Mutti, Xiaoqun Niu, Xiulin Jiang

**Affiliations:** ^1^Department of Neurosurgery, The Second Affiliated Hospital of Kunming Medical University, Kunming, China; ^2^Institute for Ecological Research and Pollution Control of Plateau Lakes, School of Ecology and Environmental Science, Yunnan University, Kunming, China; ^3^Hematology and Rheumatology Department, The Pu’er People’s Hospital, Pu’er, China; ^4^Department of Cardiovascular Surgery, The First People’s Hospital of Yunnan Province, Kunming, China; ^5^Sbarro Institute for Cancer Research and Molecular Medicine, Center for Biotechnology, College of Science and Technology, Temple University, Philadelphia, PA, United States; ^6^Department of Respiratory Medicine, Second Hospital of Kunming Medical University, Kunming, China; ^7^Kunming College of Life Science, University of Chinese Academy of Sciences, Beijing, China

**Keywords:** lncRNA, lung adenocarcinoma, prognosis biomarker, immune infiltration, ceRNA, cell proliferation, cell migration

## Abstract

Lung adenocarcinoma (LUAD) is the most common histological lung cancer, and it is the leading cause of cancer-related deaths worldwide. Long noncoding RNAs (lncRNAs) have been implicated in the initiation and progression of various cancers. LncRNA-AC099850.3 is a novel lncRNA that is abnormally expressed in diverse cancer types including LUAD. However, the clinical significance, prognostic value, diagnostic value, immune role, and potential biological function of AC099850.3 LUAD remain elusive. In this study, we found that AC099850.3 was highly expressed in LUAD and associated with an advanced tumor stage, poor prognosis, and immune infiltration. Receiver operating curve analysis revealed the significant diagnostic ability of AC099850.3 (AUC=0.888). Functionally, the knockdown of AC099850.3 restrained LUAD cell proliferation and migration *in vitro*. Finally, we constructed a competitive endogenous RNAs (ceRNA) network that included hsa-miR-101-3p and 4 mRNAs (ESPL1, AURKB, BUB3, and FAM83D) specific to AC099850.3 in LUAD. Kaplan–Meier survival analysis showed that a lower expression of miR-101-3p and a higher expression of ESPL1, AURKB, BUB3, and FAM83D, were associated with adverse clinical outcomes in patients with LUAD. This finding provided a comprehensive view of the AC099850.3-mediated ceRNA network in LUAD, thereby highlighting its potential role in the diagnosis and prognosis of LUAD.

## Introduction

Non-small cell lung cancer (NSCLC) is the main histological type of lung cancer (approximately accounts for 85% of newly diagnosed cases), while lung adenocarcinoma (LUAD) is a classification of NSCLC with high morbidity and mortality (1).

As per the World Health Organization, more than one million patients per year will die due to lung cancer in 2030 (2). Radical and palliative surgery, targeted chemotherapy, molecular targeted therapy, and immunotherapy are important methods for LUAD treatments in recent years (1). However, a poor survival prognosis exists in LUAD patients for early recurrence, metastasis, and multidrug resistance (3). Therefore, it is urgently needed to discover new biomarkers, therapeutic targets, and drugs for effectively preventing the development of lung cancer.

Long non-coding RNAs (lncRNAs) are RNA molecules with a transcript length of more than 200 nt and that lack a protein-coding ability (4). Diverse lncRNAs are an abnormal expression in human cancers and may serve as potential prognostic markers for specific cancer types (5). A growing number of studies have shown that lncRNAs play vital roles in various types of cancers *via* modulating the gene expression at transcriptional, posttranscriptional, and/or translational levels (6). For example, METTL3-induced lncRNA ABHD11-AS1 promoted NSCLC progression by facilitating the Warburg effect of NSCLC (7). LncRNA MCM3AP-AS1 is found to be upregulated in LUAD and competitively binds to the miR-148a to promote lung cell proliferation, migration, and invasion through upregulating ROCK1 expression (8). In our previous study, we developed a new method called CVAA (cross-value association analysis), which functions without a normalization and distribution assumption. We applied it to large-scale pan-cancer transcriptome data generated by The Cancer Genome Atlas (TCGA) project and successfully discovered numerous new differentially expressed genes (DEGs) (9). AC099850.3 is one of these DEGs. However, the clinical significance, prognostic value, diagnostic value, immune infiltration, and potential biological function of AC099850.3 LUAD remain elusive.

In the present study, we used various databases to analyze AC099850.3 expression and its clinical significance, prognostic value, diagnostic value, and immune infiltration and determine its potential oncogenic function in LUAD. Meanwhile, cholecystokinin octapeptide (CCK8), 5-bromo-2'-dexoyuridine (BrdU), and transwell assays were used to examine the potential biological function of AC099850.3 in LUAD progression.

## Materials and Methods

### The Cancer Genome Atlas Datasets

The download transcription and clinical information of LUAD was downloaded from TCGA (https://portal.gdc.com) (10). The RNA-seq gene expression data of workflow-type fragments per kilobase of transcript per million fragments mapped (FPKM) were transformed into a TPM format and log2 transformation for further study. The timeROC analysis was used to compare the predictive accuracy of the AC099850.3 gene in LAUD.

### The Human Protein Atlas

Human Protein Atlas (HPA) (https://proteinatlas.org/) includes the normal tissue and tumor tissue protein levels of human gene expression profile information (11). In this study, the HPA database was utilized to analysis the protein expression of ESPL1, AURKB, and BUB3 in lung cancer tissues.

### Gene Set Enrichment Analysis

In the present research, we utilized the linkedomics database (http://www.linkedomics.org/login.php) in obtaining the co-expression genes of AC099850.3 in LUAD. The gene set “kegg.v6.2.symbols.gmt,” which served as a reference gene set, was downloaded from the Molecular Signatures Database (MSigDB) (http://software.broadinstitute.org/gsea/msigdb) (12–14).

### The Target Gene of miR-101-3p Predicted by Various Databases

In this study, starBase (https://starbase.sysu.edu.cn/) is a database that decodes miRNA–ceRNA, miRNA–ncRNA, and protein–RNA interaction networks from large-scale CLIP-Seq data. miRDB (http://mirdb.org/) is an online database for miRNA target prediction and functional annotations. All the targets in miRDB were predicted by a bioinformatics tool, MirTarget, which was developed by analyzing thousands of miRNA–target interactions from high-throughput sequencing experiments. miRWalk (http://zmf.umm.uni-heidelberg.de/apps) is an open-source platform that provides an intuitive interface that generates the predicted and validated miRNA-binding sites of known genes of human, mouse, rat, dog, and cow. The core of miRWalk is the miRNA target site prediction with the random forest-based approach software TarPmiR searching the complete transcript sequence including the 5’-UTR, coding region (CDS) and 3’-untranslated region (UTR), and miRGator (http://mirgator.kobic.re.kr/), a microRNA portal for deep sequencing, expression profiling and mRNA targeting. In this study, we utilized these databases to predict the targets gene of miR-101-3p. StarBase was also used to analyze the correlation between miR-101-3p expression and AC099850.3 in LUAD (15–18).

### The Cancer Therapeutics Response Portal (CTRP) Database

CTRP (http://portals.broadinstitute.org/ctrp/), includes the links genetic, lineage, and other cellular features of cancer cell lines to small-molecule sensitivity with the goal of accelerating the discovery of patient-matched cancer therapeutics. In this study, we used the CTRP database to examine the correlation between ESPL1, AURKB, BUB3, and FAM83D and the sensitivity of various drugs.

### Cancer Therapeutics Response Portal

Cancer Therapeutics Response Portal (http://www.broadinstitute.org/ctrp) links genetic, lineage, and other cellular features of cancer cell lines to small-molecule sensitivity with the goal of accelerating the discovery of patient-matched cancer therapeutics. In this study, we used this database to determine the correlation between ESPL1, AURKB, BUB3, and FAM83D expression and drug sensitivity.

### Cell Culture Conditions

Lung cancer cell lines including one human normal bronchial epithelial cell (BEAS-2B) and 3 human LUAD cells (H358, H1975, and A549 cells) were purchased from the Chinese Academy of Sciences Cell Bank (CASCB, Shanghai, China), and cultured in RPMI 1640 medium (Corning) including 10% fetal bovine serum and 1% penicillin/streptomycin at 37°C in an atmosphere containing 95% air and 5% CO_2_.

### SiRNA and Transfection

The siRNA for AC099850.3 and the matched negative controls was designed and synthesized by RiboBio (Guangzhou, China). The Lipofectamine™3000 reagent (Invitrogen, USA) was used to transfect siRNA according to the manufacturer’s instructions. The primer used in this study is as follows: AC099850.3 siRNA#1: GCTCTGTCACCCAGGCTG.

### Quantitative Real-Time PCR

The quantitative real-time PCR (qRT-PCR) assay was performed as documented (19). The primer sequences are listed as follows: AC099850.3-F: CAGGTTCAAGTAACTGGGAC, AC099850.3-R: AATCTCTGAAGTCCATAGC; β-actin-F: CTTCGCGGGCGACGAT, β-actin-R: CCATAGGAATCCTTCTGACC. The expression quantification was obtained with the 2−ΔΔCt method.

### Cholecystokinin Octapeptide (CCK8) Assays

Cell viability and growth were determined using CCK8 assays in 96-well plates. Cells were transfected with the relevant plasmid culturing for 48 h, followed by incubation with 8 μl of CCK8 for 4 h. Absorbance was read at 450 nm using a spectrophotometer.

### 5-bromo-2'-dexoyuridine (BrdU) Incorporation Assay

For BrdU incorporation assay, a total of 3 X 104 indicated cells were plated. Approximately 20 min before fixation, indicated cells were pre-treated with 10 μM BrdU (Abcam, ab142567, 1:100) at 37°C, followed by fixation with 4% PFA and then incubation with a BrdU primary antibody overnight at 4°C. 4’,6-diamidino-2-phenylindole (DAPI) was used to stain the cell nuclei. The slides were then further stained by a secondary antibody.

### Statistical Analyses

The significance of the data between two experimental groups was determined by Student’s t-test, and multiple group comparisons were analyzed by one-way ANOVA. P < 0.05 (*), P < 0.01 (**), and P < 0.001 (***) were significant.

## Results

### AC099850.3 Expression in Lung Adenocarcinoma

**Figure 1:**
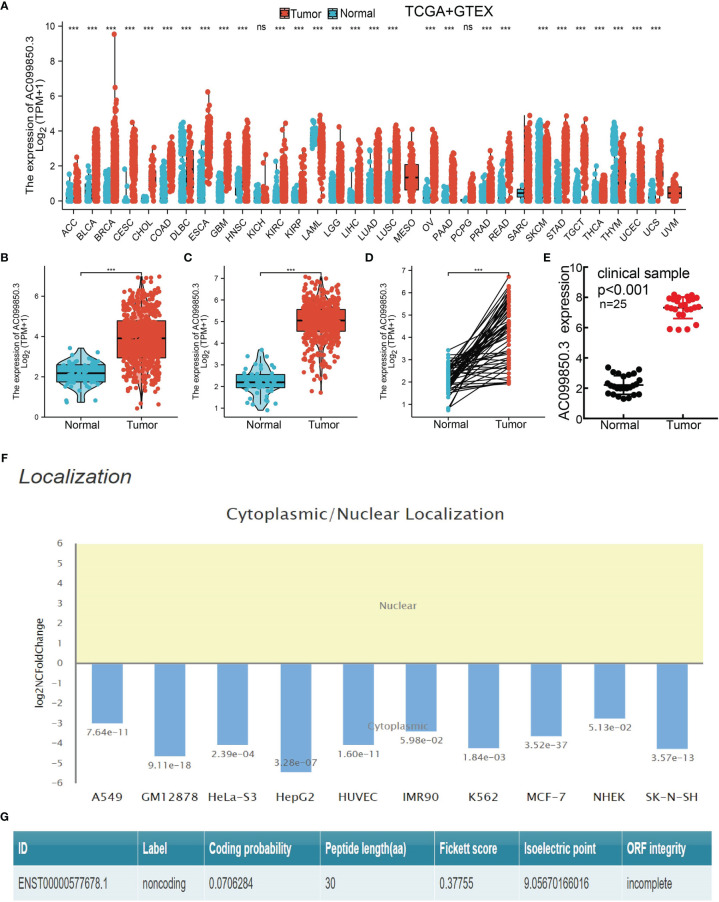
AC099850.3 was overexpressed in LUAD. **(A)** The expression of AC099850.3 in pan-cancer examined by TCGA. **(B–D)** AC099850.3 was overexpressed in LUAD and LUSC examined by TCGA. **(E)** Relative AC099850.3 expression detected by RT-qPCR in 25 paired lung cancer and noncancerous tissues. **(F)** The subcellular distribution of AC099850.3 in A549 cells was determined by annolnc2 database. **(G)** The coding probability of AC099850.3 was assessed by CPC database (NS: P > 0.05, ***P < 0.001).

We evaluated the AC099850.3expression level in pan-cancer utilizing the TCGA database. Based on the best cutoff score, we found that compared with paracancerous tissue, the AC099850.3 expression level was significantly increased in 25 types of cancer tissue (**Figure 1A**). To examine AC099850.3 expression in LUAD, we analyzed AC099850.3 expression data in TCGA-LUAD and uncovered that AC099850.3 was highly expressed in 535 tumor tissues than 59 normal prostate tissues in LUAD and upregulated in 502 tumor tissues than 49 normal prostate tissues in lung squamous cell carcinoma (LUSC) (**Figures 1B, C**). Furthermore, we demonstrated that AC099850.3 was highly expressed in 59 pairs of LUAD cancer samples than matched adjacent normal samples in TCGA data (**Figure 1D**). To further verify the expression of AC099850.3 in lung cancer tissues, we conducted a qRT-PCR assay to examine AC099850.3 expression in 25 pairs of lung cancer tissues and adjacent noncancerous tissues and found significantly higher AC099850.3 expression in lung cancer tissues than in adjacent normal tissues (**Figure 1E**). Finally, we also confirmed that AC099850.3 mainly existed in the cytoplasm of lung cells and has no protein-coding potential (**Figures 1F, G**).

### Relationship Between AC099850.3 Expression and Clinical Characteristics in Lung Adenocarcinoma

**Figure 2:**
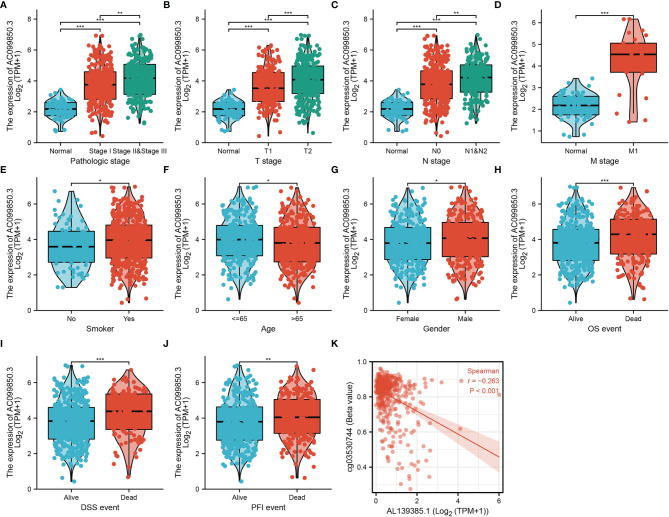
Clinical significance of AC099850.3 in LUAD. **(A–J)** Correlation between AC099850.3 expression and clinical parameters, including the pathological stage, TNM stage, smoking, age, gender, OS event, DSS event, and PFS event. **(K)** AC099850.3 expression is negatively correlated with mean AC099850.3 promoter methylation levels in TCGA LUAD cohort. *P < 0.05, **P < 0.01, and ***P < 0.001.

In order to evaluate the correlation of the AC099850.3 expression level with clinical features, we analyzed TCGA-LUAD data and found that AC099850.3 expression was significantly associated with the advanced pathological stage, tumor-node-metastasis (TNM) stage, smoking, age, gender, overall survival (OS) event, disease-free survival (DFS) event, and progression-free survival (PFS) event (**Figures 2A**–**J** and **Table 1**). Logistic regression analysis results also suggested that AC099850.3 expression was correlated with the pathological stage and TNM stage in LUAD patients (**Table 2**). A growing body of evidence confirmed that DNA methylation plays a central role in gene expression regulation and cancer progression. Therefore, we decided to explore the potential correlation between DNA methylation and AC099850.3 expression. Results showed a negative correlation between AC099850.3 expression and its diverse DNA methylation sites (**Figure 2K**).

**Table 1:** Correlation between AC099850.3 expression and clinicopathologic features in the TCGA-LUAD cohort.

Characteristic	Low expression of AC099850.3	High expression of AC099850.3	p
n	267	268	
T stage, n (%)			0.032
T1	102 (19.2%)	73 (13.7%)	
T2	129 (24.2%)	160 (30.1%)	
T3	26 (4.9%)	23 (4.3%)	
T4	8 (1.5%)	11 (2.1%)	
N stage, n (%)			0.001
N0	191 (36.8%)	157 (30.3%)	
N1	37 (7.1%)	58 (11.2%)	
N2	26 (5%)	48 (9.2%)	
N3	1 (0.2%)	1 (0.2%)	
M stage, n (%)			0.013
M0	179 (46.4%)	182 (47.2%)	
M1	8 (2.1%)	17 (4.4%)	
Age, meidan (IQR)	67 (60, 73.75)	65 (58, 72)	0.053

**Table 2:** Logistic regression analysis of correlation between AC099850.3 expression and clinical pathological characteristics.

Characteristics	Total (N)	Odds Ratio (OR)	P-value
T stage (T2&T3&T4 vs. T1)	532	1.663 (1.156-2.402)	0.006
N stage (N1&N2&N3 vs. N0)	519	2.034 (1.402-2.969)	<0.001
M stage (M1 vs. M0)	386	2.090 (0.905-5.235)	0.0095
Pathologic stage (Stage III&Stage IV vs. Stage I&Stage II)	527	1.861 (1.214-2.884)	0.005
Smoker (Yes vs. No)	521	1.626 (0.993-2.697)	0.056

### Analysis of the Diagnostic and Prognostic Values of AC099850.3 in Lung Adenocarcinoma

**Figure 3:**
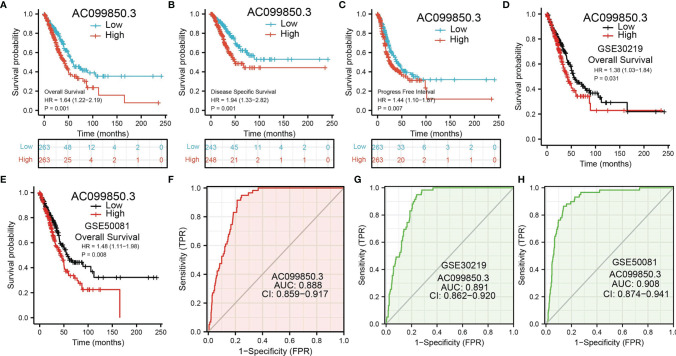
ROC and Kaplan–Meier curves of AC099850.3 **(A–C)** Kaplan–Meier survival curves showed that LUAD patients with high AC099850.3 expression exhibited poor OS, disease-specific survival, and PFS of AC099850.3 in LUAD determined by TCGA-LUAD dataset. **(D, E)** Examination of the prognosis values of AC099850.3 in lung cancer by GEO datasets. **(F–H)** ROC curves were used to determine the diagnostic value of AC099850.3 in LUAD.

**Figure 4:**
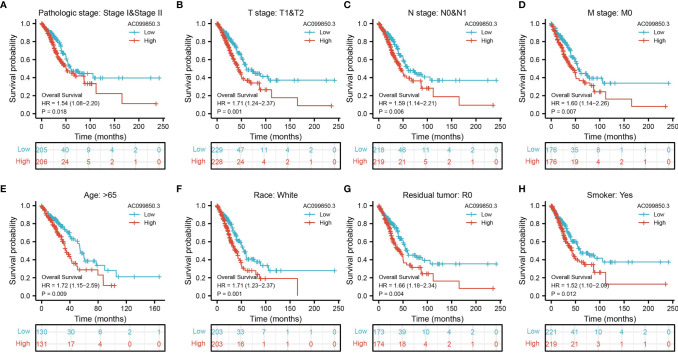
The OS of AC099850.3 based on diverse subgroup. **(A–H)** The OS of AC099850.3 based on diverse subgroup, including the stage I and II, T1 and T2, N0 and N1, M0, age >65, White, residual tumor: R0, and smoker.

Kaplan–Meier analysis results showed that increased AC099850.3 expression was significantly associated with poor OS, DFS, and PFS in patients with LUAD (**Figures 3A**–**C**). The above results were also validated by the GEO dataset (**Figures 3D, E**). Furthermore, we also examined the diagnostic value of AC099850.3 in distinguishing LUAD samples from normal lung cancer; ROC (receiver operating characteristic) curve analysis results suggested that the AUC value of AC099850.3 is 0.888 (**Figure 3F**). These results were also validated by the GEO dataset (**Figures 3G, H**). These results confirmed that AC099850.3 may be a promising biomarker for differentiating LUAD tissue from normal lung tissue. Moreover, OS analysis was conducted to determine the prognostic significance of AC099850.3 in different subgroups of LUAD patients stratified by stage I and II, T1 and T2, N0 and N1, M0, Age > 65, White, residual tumor: R0, and smoker. Results indicated that an increased AC099850.3 level is associated with poor prognosis (**Figures 4A**–**H**).

### Univariate and Multivariate Cox Regression Analyses of Different Parameters on Overall Survival

We performed univariate Cox regression analysis in the TCGA-LUAD cohort to determine whether the AC099850.3 expression level and pathologic stage might be valuable prognostic biomarkers. Univariate Cox regression analysis results show that a higher expression of AC099850.3, the pathologic stage, and TNM stage were associated with OS, DSS, and PFS in LUAD patients. To ascertain whether the AC099850.3 expression level could be an independent prognostic factor for patients with LUAD, multivariate Cox regression analysis was performed. We confirmed that the upregulation of AC099850.3 was a significant independent prognostic factor in the TCGA-LUAD cohort that was directly correlated with adverse OS, DSS, and PFS (**Tables 3**–**5**).

**Table 3:** Univariate and multivariate Cox regression analyses of different parameters on OS.

Characteristics	Total (N)	Univariate analysis		Multivariate analysis	
		Hazard ratio (95% CI)	P-value	Hazard ratio (95% CI)	P-value
Pathologic stage	518				
Stage I&Stage II	411				
Stage III&Stage IV	107	2.664 (1.960-3.621)	<0.001	6.213 (2.219-17.397)	<0.001
T stage	504				
T1	175				
T2&T3	329	1.658 (1.175-2.341)	0.004	1.675 (1.070-2.624)	0.024
N stage	510				
N0&N1	437				
N2&N3	73	2.321 (1.631-3.303)	<0.001	0.423 (0.148-1.212)	0.109
M stage	377				
M0	352				
M1	25	2.136 (1.248-3.653)	0.006	0.351 (0.110-1.117)	0.076
Smoker	512				
No	72				
Yes	440	0.894 (0.592-1.348)	0.591		
AC099850 3	526	1.223 (1.093-1.368)	<0.001	1.150 (1.001-1.320)	0.049

**Table 4:** Univariate and multivariate Cox regression analyses of different parameters on DFS.

Characteristics	Total (N)	Univariate analysis		Multivariate analysis	
		Hazard ratio (95% CI)	P-value	Hazard ratio (95% CI)	P-value
Pathologic stage	483				
Stage I&Stage II	389				
Stage III&Stage IV	94	2.436 (1.645-3.605)	<0.001	2.307 (0.447-11.894)	0.318
T stage	473				
T1	168				
T2&T3	305	1.815 (1.169-2.819)	0.008	1.727 (0.980-3.044)	0.059
N stage	475				
N0&N1	410				
N2&N3	65	1.971 (1.247-3.115)	0.004	0.913 (0.181-4.600)	0.912
M stage	344				
M0	323				
M1	21	2.455 (1.269-4.749)	0.008	1.105 (0.219-5.581)	0.904
Smoker	477				
No	69				
Yes	408	1.040 (0.602-1.796)	0.889		
AC099850 3	491	1.305 (1.129-1.508)	<0.001	1.245 (1.039-1.491)	0.017

**Table 5:** Univariate and multivariate Cox regression analyses of different parameters on PFS.

Characteristics	Total (N)	Univariate analysis		Multivariate analysis	
		Hazard ratio (95% CI)	P-value	Hazard ratio (95% CI)	P-value
Pathologic stage	518				
Stage I&Stage II	411				
Stage III&Stage IV	107	1.513 (1.105-2.071)	0.010	1.483 (1.056-2.081)	0.023
T stage	504				
T1	175				
T2&T3	329	1.923 (1.407-2.629)	<0.001	1.767 (1.287-2.427)	<0.001
N stage	510				
N0&N1	437				
N2&N3	73	1.325 (0.914-1.919)	0.137		
M stage	377				
M0	352				
M1	25	1.513 (0.855-2.676)	0.155		
Smoker	512				
No	72				
Yes	440	0.968 (0.658-1.426)	0.870		
AC099850 3	526	1.208 (1.091-1.339)	<0.001	1.154 (1.035-1.287)	0.010

### KEGG Enrichment Analysis for AC099850.3 in Lung Adenocarcinoma

**Figure 5:**
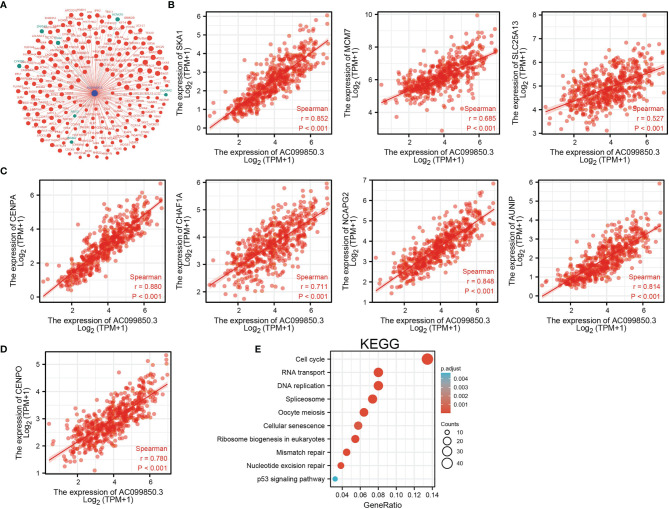
KEGG enrichment analysis of AC099850.3. **(A)** The gene–gene interaction network of AC099850.3 in LUAD. **(B–D)** Genes that were significantly positively correlated with AC099850.3 expression in LUAD based on our TCGA-LUAD data. **(E)** KEGG enrichment analysis of AC099850.3.in LUAD.

**Figure 6:**
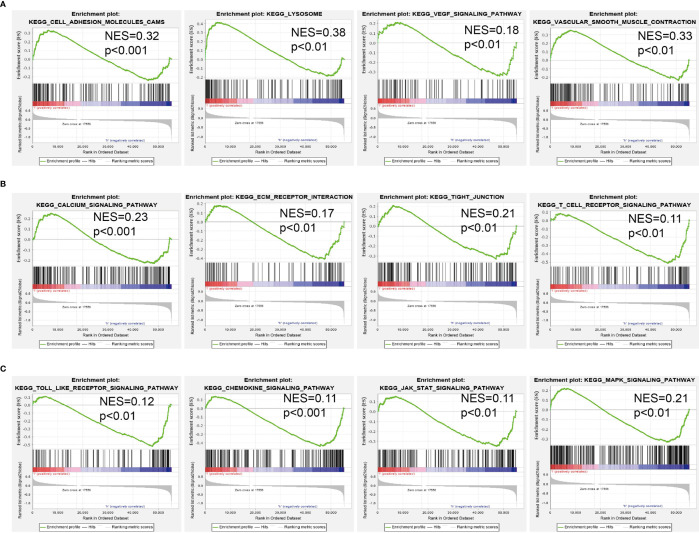
GSEA Identification of AC099850.3 related signaling pathways. **(A–C)** Identification of AC099850.3-related signaling pathways by GSEA software.

To investigate the potential biological function of AC099850.3 in the progression of LUAD, we performed KEGG enrichment analysis on genes that were significantly positively correlated with AC099850.3 expression based on TCGA-LUAD data (**Figures 5A**–**D** and **Supplementary Table 1**); the genes were significantly enriched in many pathways involved in cancer progression, including the cell cycle, DNA replication, cellular senescence, and p53 signaling pathway (**Figure 5E**). Gene set enrichment analysis (GSEA) also showed that pathways, including cell adhesion molecules, lysosomes, the vascular endothelial growth factor (VEGF) signaling pathway, vascular muscle contraction, calcium signaling pathway, extracellular matrix (ECM) receptor interaction, tight junction, T-cell receptor signaling pathway, Toll-like signaling pathway, chemokine signaling pathway, JAK/STAT signaling pathway, and MAPK signaling pathway, were significantly enriched in the high AC099850.3 expression group (**Figures 6A**–**C**).

### Correlation Between AC099850.3 Expression and Immune Infiltration

**Figure 7:**
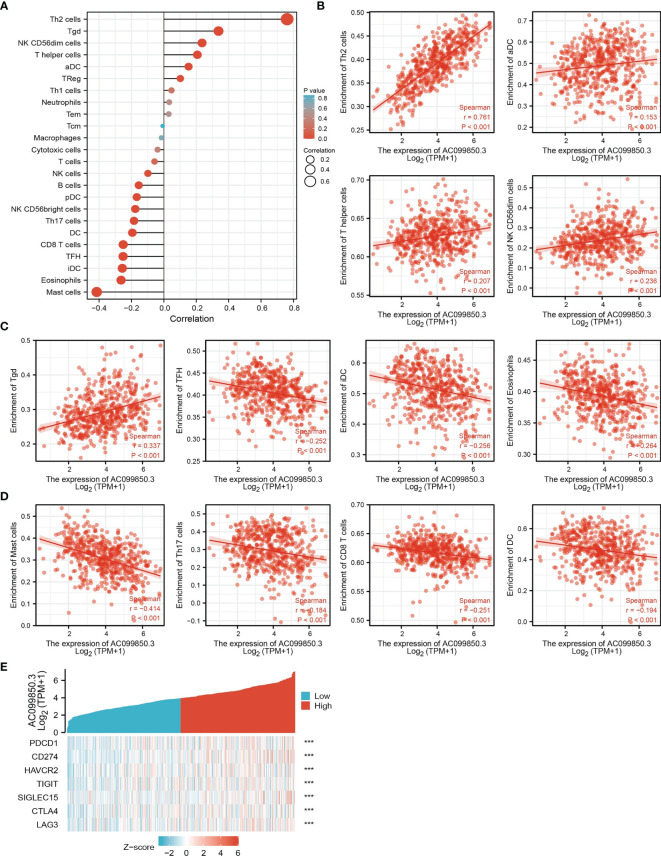
Correlation analysis of AC099850.3 expression and infiltration levels of immune cells in LUAD. **(A–D)** The correlation between AC099850.3 expression and the infiltration levels of 24 immune cells in LUAD. **(E)** The correlation between AC099850.3 expression and immune checkpoint-related genes in LUAD. ***P < 0.001.

The infiltration of immune cells has a crucial role in cancer progression (20). Therefore, we decided to explore the correlation between AC099850.3 expression and the infiltration levels of 24 types of immune cells in LUAD using the ssGSEA method. Results suggested that AC099850.3 expression in LUAD was positively correlated with the infiltration of Th2 cells, Tgd, Natural Killer (NK) CD56dim cells, T helper cells, and activated dendritic cells (aDC), and negatively correlated with the abundance of Th17 cells, DC, CD8 T cells, T follicular helper (Tfh), immature dendritic cells (iDC), eosinophils, and mast cells in LUAD (**Figures 7A**–**D**).

Considering that AC099850.3 might be the potential oncogene in LUAD, the relationship of AC099850.3 with PDCD1, CD274, HAVCR2, TIGIT, SIGLEC15, CTLA4, LAG3, and PDCD1LG2 in LUAD was assessed. As a result, we found that the expression levels of AC099850.3 had a significantly positive correlation with PDCD1, CD274, HAVCR2, TIGIT, SIGLEC15, CTLA4, and LAG3 in LUAD (**Figures 7E**). These results indicated that tumor immune escape and antitumor immunity might be involved in the AC099850.3-mediated carcinogenic processes of LUAD.

### AC099850.3-Related miRNA–mRNA Network in Lung Adenocarcinoma

**Figure 8:**
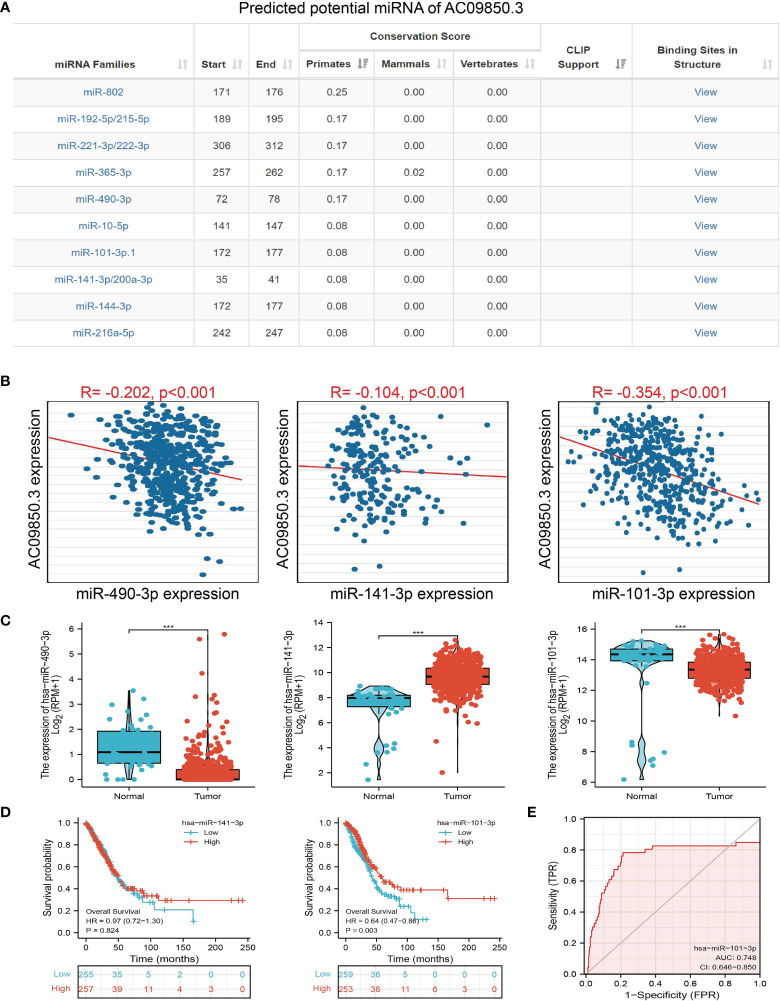
Analysis of the potential miRNAs of AC099850.3. **(A)** The potential miRNAs of AC099850.3 determined by Anbolnc2 database. **(B)** Correlations between AC099850.3 expression and miR-490-3p, miR-141-3p, and miR-101-3p in LUAD **(C)** The expression level of miR-490-3p, miR-141-3p, and miR-101-3p in LUAD **(D)** The OS of miR-141-3p, and miR-101-3p in LUAD. **(E)** ROC curve of miR-101-3p in LUAD. ***P < 0.001.

To further explore the AC099850.3-mediated downstream regulatory mechanism involved in LUAD progression, we used the Annolnc2 (http://annolnc.gao-lab.org/) database, which predicted the potential miRNAs that bind with AC099850.3; we obtained a total of 10 miRNAs (**Figure 8A**) (21). Based on the competitive endogenous RNA theory, lncRNA should be positively correlated with mRNA and negatively correlated with miRNA. Among all the 10 miRNAs, has-miR-490-3p, has-miR-141-3p, and has-miR-101-3p were negatively correlated with AC099850.3 in LUAD (**Figure 8B**). We also found that has-miR-101-3p and has-miR-490-3p were decreased in LUAD and only the has-miR-101-3p lower expression was correlated with poor prognosis in patients with LUAD (**Figures 8C, D**). ROC curve analysis showed that the AUC value of has-miR-101-3p is 0.748 (**Figure 8E**). Therefore, we selected has-miR-101-3p in conducting downstream analysis.

### Identification of the Potential Downstream Target of AC099850.3/miR-101-3p in Lung Adenocarcinoma

**Figure 9:**
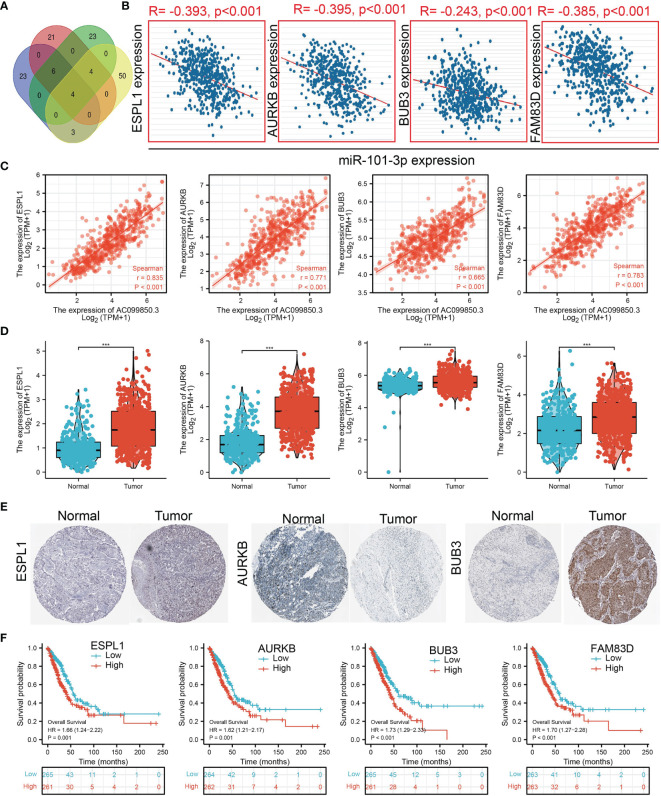
Analysis of the potential mRNAs of AC099850.3/miR-101-3p. **(A)** Identifying ESPL1, AURKB, BUB3, and FAM83D as the downstream target of miRNA-101-3p using various datasets. **(B)** Correlations between miRNA-101-3p expression and ESPL1, AURKB, BUB3, and FAM83D in LUAD. **(C)** Correlations between AC099850.3 expression and ESPL1, AURKB, BUB3, and FAM83D in LUAD. **(D, E)** The RNA and protein level of ESPL1, AURKB, BUB3, and FAM83D in LUAD. **(F)** The overall survival of GALNT3, CYCS, EIF5A, and ITGB4 in LUAD. ***P < 0.001.

**Figure 10:**
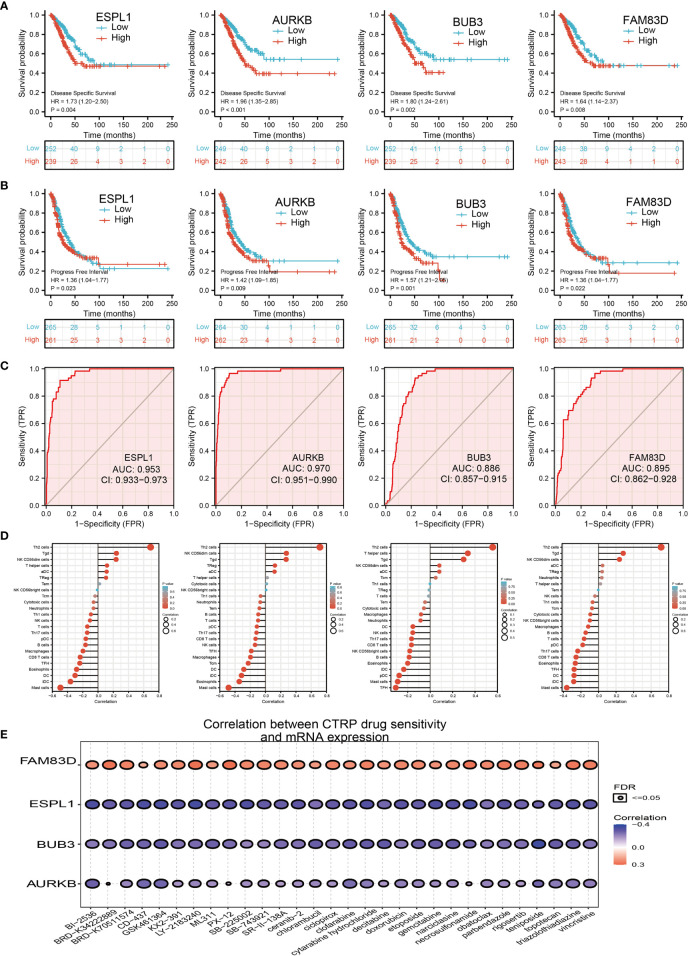
Analysis of the GALNT3, CYCS, EIF5A, and ITGB4 expression and the immune level of correlation. **(A, B)** The DFS and PFS of ESPL1, AURKB, BUB3, and FAM83D in LUAD. **(C)** ROC curve of ESPL1, AURKB, BUB3, and FAM83D in LUAD. **(D)** The correlation between ESPL1, AURKB, BUB3, and FAM83D expression and the infiltration levels of 24 immune cells in LUAD. **(E)** The correlation between ESPL1, AURKB, BUB3, and FAM83D expression and the sensitivity of diverse drug determined by CTRP database.

We further investigated the target genes of miR-101-3p that play critical roles in the progression of LUAD. Firstly, we predicted the target in StarBase, miRDB, miRWalk, and miRGator (15–18) (**Figure 9A**). According to the prediction of target genes, we found that only 4 genes (ESPL1, AURKB, BUB3, and FAM83D) were negatively correlated with miR-101-3p expression in LUAD (**Figure 9B**). Importantly, the expression of ESPL1, AURKB, BUB3, and FAM83D was positively correlated with that of AC099850.3 in LUAD (**Figure 9C**). Furthermore, we employed the TCGA and HPA databases to explore the expression level and prognosis in LUAD. We found that the mRNA and protein of ESPL1, AURKB, BUB3, and FAM83D were significantly increased in LUAD and associated with OS, DSS, and PFS in patients with LUAD (**Figures 9D**–**F** and **10A, B**). The ROC curve was utilized to examine the diagnostic value of ESPL1, AURKB, BUB3, and FAM83D in LUAD, the AUC (area under the curve) of which were 0.953, 0.970, 0.886, and 0.895, respectively (**Figure 10C**), suggesting that ESPL1, AURKB, BUB3, and FAM83D were potential prognostic and diagnostic biomarkers in LUAD. Finally, we used the ssGSEA method to determine the correlations between ESPL1, AURKB, BUB3, and FAM83D and 24 types of tumor-infiltrating immune cells. Results confirmed that ESPL1, AURKB, BUB3, and FAM83D expression were positively correlated with the cell infiltration of Th2 cells and negatively correlated with the cell infiltration of mast cells (**Figure 10D**). We also confirmed that ESPL1, AURKB, and BUB3 expression was negative, FAM83D was positively correlated with sensitivity of various drugs based on the CTRP database (**Figure 10E**).

### AC099850.3 Modulates Cell Proliferation and Migration of Lung Adenocarcinoma Cells

**Figure 11:**
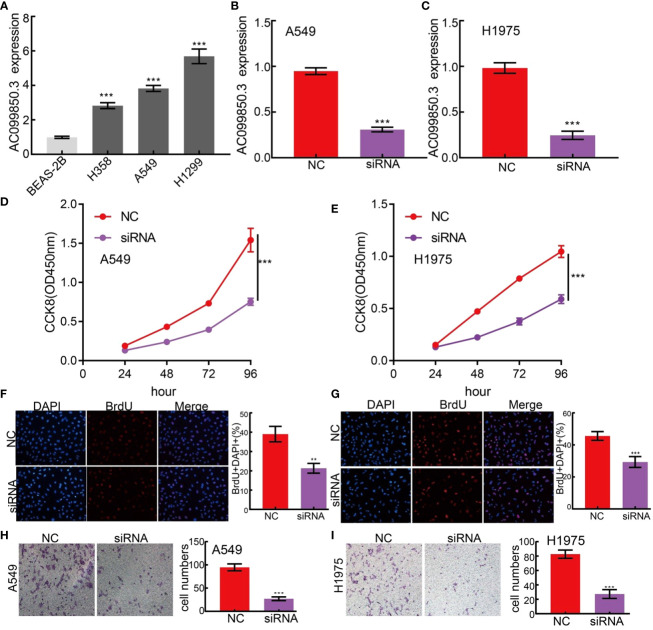
lncRNA-AC099850.3 modulates LUAD cell proliferation and migration *in vitro*. **(A)** The relative expression level of AC099850.3 in lung adenocarcinoma cancerous cell lines, including H358, H1975 and A549 examined by Real-time RT-PCR, compared to normal human bronchial epithelial cell line: BEAS-2B. **(B, C)** Establishment of AC099850.3 knockdown cell lines in A549 and H1975 verified by Real-time RT-PCR **(D–G)** Knockdown of lncRNA AC099850.3 significantly inhibits cell proliferation as measured by CCK8 and BrdU assay. **(H, I)** knockdown of lncRNA AC099850.3 dramatically inhibits LUAD cells migration ability examined by transwell assay. ***P < 0.001. NC, Negative control; siRNA, AC099850.3 siRNA.

The above studies indicated that AC099850.3 expression was distinctly increased in LUAD tissues, and AC099850.3 might influence the progression in LUAD. To further investigate the biological role of AC099850.3 in LUAD, we first confirmed that the expression of AC099850.3 was significantly upregulated in H358, H1975, and A549 lung cancer cell lines (**Figure 11A**). Moreover, a specific siRNA for AC099850.3 was used to the knockdown of AC099850.3 expression. The knockdown efficiencies in transformed cell lines were detected by qRT–PCR analysis (**Figures 11B, C**). It was shown that the depletion of AC099850.3 inhibited the proliferative capacity of LUAD cells and markedly decreased the ratio of BrdU-positive cells (**Figures 11D**–**G**). Moreover, the transwell assay suggested that the depletion of AC099850.3 significantly inhibited the cell migration of LUAD (**Figures 11H, I**). Taken together, these data suggest that AC099850.3 is functionally important in regulating LUAD progression.

## Discussion

Increasing evidence demonstrated the functional and clinical role of lncRNAs involved in the progression (7, 22). For example, it has been shown that increased lncRNA AC079630.4 expression is correlated with the progression and prognosis in lung cancer (23). Previous studies indicated that lncRNAs have clinical predictor value in several tumors (24). For instance, Wang et al. found that linc8087 was downregulated in NSCLC and its lower expression was associated with adverse prognosis in patients with NSCLC (25).

In the current study, we found that suggested that increased AC099850.3 expression was correlated with adverse OS, DFS, and PFS in patients with LUAD. Additionally, ROC curve analysis confirmed that the AUC value of AC099850.3 is 0.888. Results suggested that AC099850.3 may be a promising biomarker for differentiating LUAD tissue from normal lung tissue. Multivariate analysis indicated that AC099850.3 expression was an independent prognostic indicator for the OS, DFS, and PFS of LUAD patients.

Previous studies reported that lncRNA plays an important role the EMT and cell cycle (20, 22). For example, it has been confirmed that lncRNA-JPX promoted the cell proliferation and migration by sponging miR-33a-5p to increased Twist1expression (19). In this study, we investigated the underlying mechanisms through which AC099850.3 affected the progression of LUAD. We found that AC099850.3 was significantly associated with the cell adhesion molecules, lysosomes, VEGF signaling pathway, vascular muscle contraction, calcium signaling pathway, ECM receptor interaction, tight junction, T-cell receptor signaling pathway, Toll-like signaling pathway, chemokine signaling pathway, JAK/STAT signaling pathway, and MAPK signaling pathway.

It has been confirmed that lncRNA plays a central role in facilitating tumor progression and immune escape (26). For example, lncRNA GATA3-AS1 promoted cancer progression and immune escape in BRCA *via* the stabilization of PD-L1 (26). In this finding, we demonstrated that AC099850.3 expression was positively correlated with the infiltration of Th2 cells, Tgd, NK CD56dim cells, T helper cells, and aDC and negatively correlated with the abundance of Th17 cells, DC, CD8 T cells, TFH, iDC, eosinophils, and mast cells in LUAD.

The lncRNAs could target a series of miRNAs, and the lncRNA–miRNA network reveals a crucial role in tumors (27, 28). In this study, the potential AC099850.3-related miRNAs were probed. Among these miRNAs, miR-101-3p was selected to verify the interaction with AC099850.3. Several studies demonstrated that miR-101-3p was downregulated in LUAD and could inhibit tumor cell proliferation and invasion by targeting BIRC5 in LUAD (29). Moreover, it has been shown that miR-101-3p, *via* targeting EZH2, plays a tumor suppressor role in renal cell carcinoma (30). In our study, we found that miR-101-3p was downregulated in LUAD and its lower expression is correlated with adverse clinical outcomes in patients.

We also utilized various databases to identify the potential target genes of AC099850.3/miRNA-101-3p in LUAD, including ESPL1, AURKB, BUB3, and FAM83D. Subsequent expression and prognosis analysis confirmed that ESPL1, AURKB, BUB3, and FAM83D were significantly greater in LUAD tissues than those in the normal LUAD tissues. Immunohistochemical analysis revealed that the expression of ESPL1, AURKB, and BUB3 proteins was higher in LUAD tissues than that in the lung tissues. In addition, the upregulation of ESPL1, AURKB, BUB3, and FAM83D expression was associated with poor prognosis in patients with LUAD.

It has been confirmed that AURKB was increased in LUAD and its higher expression was negatively associated with OS, post-progression survival, and first progression (31). Another study found that AURKB may be a target in NSCLC with acquired resistance to anti-EGFR therapy (32). Our findings are consistent with previous studies. In conclusion, this finding provided a possible comprehensive view of the AC099850.3-mediated ceRNA network in LUAD, thereby highlighting its potential role in diagnosis and therapy. Finally, we uncovered that AC099850.3 was significantly upregulated in NSCLC cell lines and the depletion of AC099850.3 markedly inhibited cell proliferation and migration in LUAD.

This study improves our understanding of the correlation between AC099850.3 and LUAD, but some limitations still exist. First, although we explored the correlation between AC099850.3 and immune infiltration in LUAD patients, there is a lack of experiments to validate the function of AC099850.3 in the tumor microenvironment regulation of LUAD. Second, we uncovered that the knockdown of AC099850.3 inhibits the cell proliferation and cell migration of LUAD. However, the potential molecular mechanisms of AC099850.3 in cancer progression need to be explored in further studies.

## Conclusion

Our data confirmed that AC099850.3 can serve as a promising diagnostic and prognostic biomarker for LUAD patients.

## Data Availability Statement

The original contributions presented in the study are included in the article/**Supplementary Material**. Further inquiries can be directed to the corresponding authors.

## Author Contributions

XC, JG, FZ, and WR designed this work and performed related assay and analyzed the data. JP, LM, XN, and XJ supervised and wrote the manuscript. All authors have read and approved the final version of the manuscript.

## Funding

This study was supported by Applied Basic Research Project of Yunnan Provincial Science and Technology Department and Kunming Medical University, No.2020001AY070001-117 and the Open Project of The First People’s Hospital of Yunnan Province Clinical Medicine Center (2021LCZXXF‐XZ03).

## Conflict of Interest

The authors declare that the research was conducted in the absence of any commercial or financial relationships that could be construed as a potential conflict of interest.

## Publisher’s Note

All claims expressed in this article are solely those of the authors and do not necessarily represent those of their affiliated organizations, or those of the publisher, the editors and the reviewers. Any product that may be evaluated in this article, or claim that may be made by its manufacturer, is not guaranteed or endorsed by the publisher.
