# Correction: Over-expression of long non-coding RNA-AC099850.3 correlates with tumor progression and poor prognosis in lung adenocarcinoma

**DOI:** 10.3389/fonc.2025.1641207

**Published:** 2025-07-21

**Authors:** Xi Chen, Jishu Guo, Fan Zhou, Wenjun Ren, Jun Pu, Luciano Mutti, Xiaoqun Niu, Xiulin Jiang

**Affiliations:** ^1^ Department of Neurosurgery, The Second Affiliated Hospital of Kunming Medical University, Kunming, China; ^2^ Institute for Ecological Research and Pollution Control of Plateau Lakes, School of Ecology and Environmental Science, Yunnan University, Kunming, China; ^3^ Hematology and Rheumatology Department, The Pu’er People’s Hospital, Pu’er, China; ^4^ Department of Cardiovascular Surgery, The First People’s Hospital of Yunnan Province, Kunming, China; ^5^ Sbarro Institute for Cancer Research and Molecular Medicine, Center for Biotechnology, College of Science and Technology, Temple University, Philadelphia, PA, United States; ^6^ Department of Respiratory Medicine, Second Hospital of Kunming Medical University, Kunming, China; ^7^ Kunming College of Life Science, University of Chinese Academy of Sciences, Beijing, China

**Keywords:** lncRNA, lung adenocarcinoma, prognosis biomarker, immune infiltration, ceRNA, cell proliferation, cell migration

## Error in Figure

In the published article, there was an error in **Figure 11G** as published. The representative picture of BRDU, H1975 cells was presented incorrectly. This occurred primarily because there were too many pictures taken with the microscope on the disc. Confusing duplicate document numbers led us to upload the wrong image. The corrected **Figure 11G** appears below.

**Figure 11:**
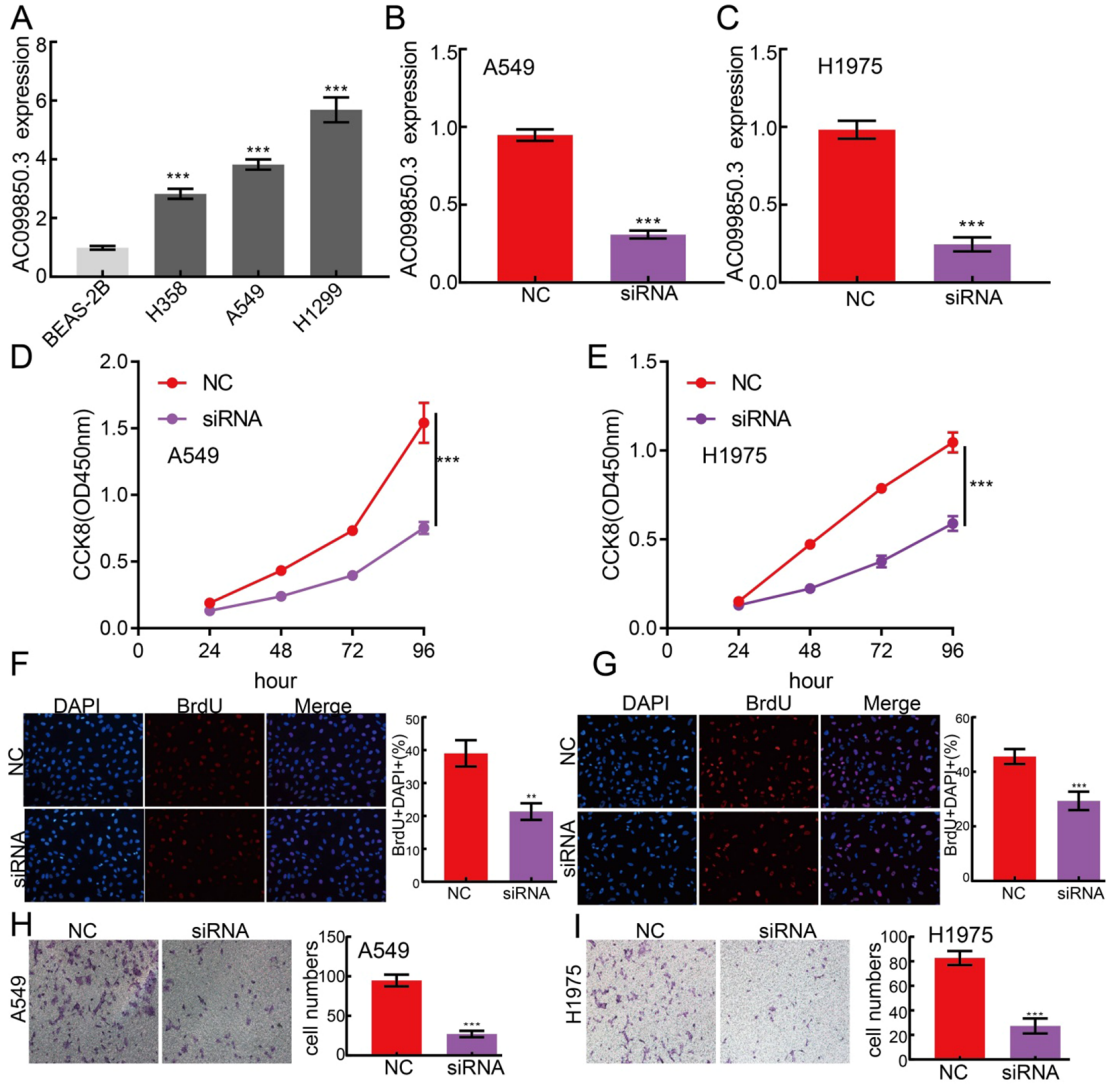
lncRNA-AC099850.3 modulates LUAD cell proliferation and migration *in vitro*. **(A)** The relative expression level of AC099850.3 in lung adenocarcinoma cancerous cell lines, including H358, H1975 and A549 examined by Real-time RT-PCR, compared to normal human bronchial epithelial cell line: BEAS-2B. **(B, C)** Establishment of AC099850.3 knockdown cell lines in A549 and H1975 verified by Real-time RT-PCR **(D–G)** Knockdown of lncRNA AC099850.3 significantly inhibits cell proliferation as measured by CCK8 and BrdU assay. **(H, I)** knockdown of lncRNA AC099850.3 dramatically inhibits LUAD cells migration ability examined by transwell assay. ***P < 0.001. NC, Negative control; siRNA, AC099850.3 siRNA.

The authors apologize for this error and state that this does not change the scientific conclusions of the article in any way. The original article has been updated.

